# Group psychological intervention for postnatal depression: a nested qualitative study with British South Asian women

**DOI:** 10.1186/s12905-015-0263-5

**Published:** 2015-11-25

**Authors:** Yumna Masood, Karina Lovell, Farah Lunat, Najia Atif, Waquas Waheed, Atif Rahman, Rahena Mossabir, Nasim Chaudhry, Nusrat Husain

**Affiliations:** Cumbria Partnership Foundation Trust, Garburn House, Westmoreland General Hospital, Burton Road, Kendal, LA97RG UK; The School of Nursing, Midwifery and Social Work, The University of Manchester, Jean McFarlane Building, University Place, Oxford Road, Manchester, M13 9PL UK; Lancashire Care NHS Foundation Trust, Whalley Road, Accrington, BB5 5AD UK; Centre for Primary Care, Academic Health Sciences Centre, University of Manchester, Oxford Road, Manchester, M13 9PL UK; Institute of Psychology, Health & Society, University of Liverpool, The Waterhouse Building, Dover Street, Liverpool, Merseyside L3 5DA UK; Child Mental Health Unit, Alder Hey Children’s NHS Foundation Trust, Mulberry House, Eaton Road, Liverpool, L12 2AP UK; Institute of Brain, Behaviour, and Mental Health, University of Manchester, Jean McFarlane Building, Oxford Road, Manchester, M13 9PL UK

**Keywords:** Cognitive behavioural therapy, British South Asian, Postnatal depression, Cultural adaptation

## Abstract

**Background:**

Postnatal depression affects 10–15 % of all mothers in Western societies and remains a major public health concern for women from diverse cultures. British Pakistani and Indian women have a higher prevalence of depression in comparison to their white counterparts. Research has shown that culturally adapted interventions using Cognitive Behavioural Therapy (CBT) may be acceptable and may help to address the needs of this population. The aim of this study was to assess the acceptability and overall experience of the Positive Health Programme by British South Asian mothers.

**Methods:**

This was a nested qualitative study, part of an exploratory randomized controlled trial (RCT) conducted to test the feasibility and acceptability of a culturally-adapted intervention (Positive Health Programme or PHP) for postnatal depression in British South Asian women. In-depth interviews (*N* = 17) were conducted to determine the views of the participants on the feasibility and acceptability of the intervention.

**Results:**

The participants found the intervention acceptable and experienced an overall positive change in their attitudes, behaviour, and increased self-confidence.

**Conclusions:**

The findings suggest that the culturally adapted Positive Health Programme is acceptable to British South Asian women. These results support that culturally sensitive interventions may lead to better health outcomes and overall satisfaction.

**Trial registration:**

Protocol registered on Clinicaltrials.gov NCT01838889

## Background

Postnatal depression affects approximately 8.5–11.0 % of all mothers in Western societies [1, 2]. The prevalence of perinatal depression is higher among mothers from certain ethnic minority groups [3]. This indicates ethnicity to be a significant risk factor for developing postnatal depression. Ethnicity has also been reported to be linked to poor health outcomes for both ethnic minority mothers and their infants, as reflected in higher rates of infant and maternal mortality and morbidity and lower birth-weight [4]. Part of this risk lies in the interaction of economic disadvantage with ethnic minority grouping.

Postnatal depression is a treatable disorder [5]. Antidepressants are effective, but new mothers are reluctant to take such medications whilst nursing [6]. Among psychotherapeutic interventions, Interpersonal Psychotherapy (IPT) and Cognitive Behavioural Therapy (CBT) have shown the most efficacy in the treatment of postnatal depression [6–8]. With regard to the postnatal treatment of British ethnic minority women, the National Institute of Clinical Excellence (NICE) [9] 2007 guidelines recommend that they should be provided with culturally sensitive information and treatment. However, we know from our earlier work [10, 11] that several barriers prevent access to appropriate mental health services for British South Asian women. Even where help is accessible it is not sensitive to the cultural needs of this population.

British South Asian women have higher birth rates compared to the majority white population and are considered ‘difficult to reach’ due to language and cultural barriers. Research [12, 13] indicates that British Pakistani women have high rates of postnatal depression, lack social support, experience interpersonal relationship problems [12] and linguistic and cultural barriers in accessing services [14]. Isolation and lack of social support are therefore important elements to be addressed in interventions. There is, however, little empirical evidence addressing the adaptation of evidence-based interventions to ensure their applicability to specific ethnic communities [15–18]. Recent studies evaluating a culturally-sensitive psychosocial group intervention for the treatment of depression in British Pakistani women reported an improvement in participants’ self-confidence at the end of the intervention [11, 19]. Overall, the findings indicated that social groups which had taken into consideration the sociocultural needs of British Pakistani women experiencing depression were acceptable to them.

Despite the above research, there is a clear gap in evidence for informing culturally-sensitive interventions for maternal depression in British South Asian women [18–20]. This paper provides a qualitative evaluation of a psychosocial intervention called the Positive Health Programme (PHP), which was adapted for and offered to British South Asian women experiencing postnatal depression. The programme was designed to be delivered to groups of participants using the cognitive behavioural model [21]. The manual assisted programme, tailored to the cultural needs of British Pakistani women, was developed by our group and initially feasibility tested for a PhD project [22]. It consisted of 12 weekly group sessions delivered over 3 months. The manual is organized into 9 distinct sections.

### The main trial

This paper describes the results of a post-intervention qualitative study as part of an exploratory randomized controlled trial conducted to test the feasibility and acceptability of a culturally-adapted intervention (Positive Health Programme or PHP) for postnatal depression in British South Asian women. The study was carried out across Manchester and Lancashire (UK). The recruitment (*N* = 615) and trial retention figures (70 %) highlight the ability of the research team to engage with this population. In the intention-to-treat analysis there was no significant difference between intervention and control groups for any of the depression scores at either follow-up. Following the initial analysis, another analysis was carried out based on attendance to the intervention. Completers were defined as those participants who had attended 4 or more sessions. Fifteen of the 42 patients in the intervention group did not attend any of the therapy sessions, while the remaining 27 patients attended at least 4 sessions. Overall, the mean number of sessions attended was 6.6 (SD = 5.2). The Spearman’s correlation coefficient between the number of sessions attended and reduction in the Hamilton score from baseline to follow-up 1 was 0.35 (*p* = 0.048), with greater reductions in the Hamilton score being associated with more sessions attended. Correlations between changes from baseline to follow-ups in the other variables ranged from −0.20 for EPDS at the second follow-up.

This paper describes the qualitative phase of the trial and explores the participants’ experiences of receiving the PHP.

### Aims

To assess the acceptability and overall experience of the Positive Health Programme by British South Asian mothers.

### Objectives

1. To identify the barriers faced by the British South Asian women in accessing the intervention.
2. To identify factors which facilitated participants’ engagement with the PHP.

## Methods

This study aims to explore a wide range of views and experiences of British South Asian women which quantitative methods do not often allow to be explored in detail [23]. In contrast, qualitative methods for data collection are sensitive to the unique personal experiences, perceptions, beliefs, and meanings related to individuals and therefore believed to be the most appropriate method to be employed within this study [24]. Qualitative analysis also offers an accessible and theoretically flexible approach to analysing qualitative data [24]. One of the strengths of this approach is that it enables the researcher to focus on identifying themes and patterns of behaviours [25].

### Recruitment

A total of 83 women with a confirmed diagnosis of depression using the Clinical Interview Schedule-Revised (CISR) interview [26] took part in the RCT. These women were of South Asian origin as defined by the Office of National Statistics (ONS) (ONS is the recognised national statistical institute for the UK providing official statistics), over the age of 16 years, and living with their babies. The PHP programme was offered to 42 British South Asian women who were randomly allocated to the intervention arm of the randomised controlled trial (RCT). The women were recruited between 2012 and 2013 from multiple sites in Manchester and Lancashire, including General Practices and Children Centres, and were organised into 4 groups for evaluation of the PHP. The programme was delivered at the local Sure Start children’s centre over a 12-week period by trained research staff. Of the 42 women who were randomised to the PHP group intervention, 20 were randomly selected using the random number tables and were invited for qualitative interviews. A total of 20 women were contacted for obtaining informed consent, 17 agreed to take part, whilst three could not be contacted. Five of the participating women were from the Manchester intervention group and the remaining 12 were from the Lancashire intervention group. The interviews took place at the participants’ homes as most did not have access to alternative child care arrangements.

### Procedure

In-depth interviews were conducted with the participants using a topic guide. The topic guide for this study was developed and informed by both existing literature in this area and an earlier feasibility study [22]. The key areas explored were reasons for participation, barriers to receiving the intervention, and perception of the intervention process including participants’ initial expectations and reflections. The interviews explored participants’ views on how the PHP sessions took place, the format and frequency of the sessions, the characteristics of the facilitator, the use of handouts and other session materials, and the acceptability of the delivery of the intervention. Each interview lasted approximately 45 min and was conducted in the participant’s preferred language; all the interviews were carried out in the Urdu language. Later these interviews were translated into English. The analysis and coding process was also carried out in English.

### Ethics approval

The study was approved by the North West Research Ethics Committee (10/H1005/62).

### Analysis

Interviews were transcribed verbatim, anonymised, and analysed using thematic analysis [27]. Thematic analysis is a flexible approach where, in contrast to other qualitative methods, the researcher is attached to a theoretical framework [28]. Thematic analysis is appropriate for use in a multi-method context where the qualitative element of enquiry is framed by prior work consistent with the present study which is embedded in a larger randomised controlled trial. The complete interview recordings were listened to and the corresponding transcripts were read over multiple times for familiarization [29]. A code book was maintained to keep the process of coding systematic and intact. Specific events, thoughts, and actions were coded or identified as themes on the basis of their ability to “capture something important in relation to the overall research question” ([27], p.82) by the researcher, YM. The researcher did not try to fit the codes into existing code frameworks. The coding process aimed to identify themes that were internally homogenous, externally homogenous, and had explanatory power. This involved an iterative process comparing themes against transcript data in order to ensure that the emerging structure of themes continued to be grounded only in the data set. Ultimately, the data collection process ended due to thematic saturation (where no additional themes were achieved) [28]. Participants’ responses were first analysed on a semantic level, whilst the main themes were developed at the second stage, involving latent analysis of how coherently and adequately the themes represented the meaning of the data as a whole [27]. Themes and sub-themes were reviewed between the researcher (YM) and the supervisor (KL) before the analysis was finalized.

## Results

A total of 17 women took part in the qualitative interviews; the age range was 20–45 years. The sample characteristics are described in Table 1.

**Table 1:** Sample characteristics of all participants (*n* = 17)

Participant	Age range	Generation	Ethnicity	Marital status
1	30–35	First	Pakistani	Married
2	25–30	Second	Pakistani	Married
3	20–25	First	Pakistani	Married
4	35–40	First	Pakistani	Married
5	20–25	Second	Pakistani	Married
6	30–35	Second	Pakistani	Married
7	35–40	Second	Indian	Married
8	25–30	Second	Bangladeshi	Married
9	40–45	First	Pakistani	Married
10	30–35	First	Pakistani	Married
11	40–45	Second	Pakistani	Married
12	25–30	First	Pakistan	Divorced
13	30–35	First	Indian	Married
14	30–35	First	Pakistani	Married
15	25–30	First	Pakistani	Married
10	30–35	First	Pakistani	Married
17	30–35	First	Pakistani	Married

### Motivators to participate in the Positive Health Programme (PHP)

Most of the women stated overcoming depression through a better understanding of their mental health issues, enhancing their confidence, meeting other women in similar situations, and sharing experiences as the major motivating factors for participation in the programme.

Yes, definitely! I wanted to come out of depression and wanted to meet new people because to me loneliness was the main reason of my depression. And to me, if there are other women who are lonely, they should meet up and join these groups. Because of these you get to know about other people and their situations. (ID 3)

### Barriers to attendance and commitment

The sessions were held during the mornings which most participants found convenient since it did not impact their daily chores. Furthermore they had the support of their family members.

No, there was no issues, no problems. My husband is really supportive and he allowed me to go and join these groups. (ID 3)

However, a few participants mentioned restrictions from their husbands because they could not comprehend the need for the intervention. At times the husbands were not informed about the sessions or were not told of the nature of the sessions for fear they would not allow the participants to attend.

My husband did not want me to go; he did not let me go anywhere. I had to look after my children, but he just wanted me to sit with him and talk to him. (ID 2)

My husband didn’t know that I was going to these classes. I used to be home before he came back from his work. (ID 9)

The participants considered the absence of childcare arrangements as a potential barrier to attending the sessions.

And we were offered a crèche facility; I used to take him there; otherwise it would have been really difficult for me. (ID 13)

Most of the participants did not drive and relied on family members for transport arrangements to and from the centre. Having transport expenses paid by the programme was appreciated by all the participants because it reduced the burden of transportation expenses on the participants.

Yes, there was the issue of travelling. I cannot drive and my husband was admitted to the hospital. But then they said they would pay me, so I continued with the classes. (ID 1)

Most of the participants reported difficulty in completing the between-session work due to excessive domestic responsibilities. However, all participants did realise that between-session work was important for improving their psychosocial well-being.

The homework was useful but my children were young and I couldn’t do that much. I tried but I could not do that much. (ID 11)

### Understanding of cultural and linguistic needs

Group facilitators conducting the sessions were of South Asian origin which enabled them to communicate in the participants’ first language (Urdu) resulting in increased group engagement and participation.

She spoke in Urdu and I had no problems. If she had spoken in English then I might have had some problems and I would have not even attended. (ID 3)

Some of the participants expressed concerns over not being able to read the handouts due to lack of English reading skills. This resulted in their dependence on other group members and facilitators to understand the handout content. It was suggested that the handouts be translated into the participants’ first language to make them useful for the readers.

For me it was ok. I did not have any problem; but I feel for women who could not read English handouts need to be written in Urdu. These women felt that they were depending on us or the facilitator for guidance. (ID 4)

The participants appreciated the facilitator’s knowledge and understanding of their socio-cultural dynamics which enabled the facilitators to relate to the participating women’s culturally specific issues and address them in the sessions sensitively.

Because she understood what we go through, how our culture is, and how our belief systems are. She could understand us better than anyone else. (ID 5)

Most importantly, facilitators’ skills such as the ability to listen and empathise, encouraging and non-judgemental attitude were recognized as important, facilitating open and honest discussions within the groups.

…the main thing is that she listened to all of us. There were times that we did not want to talk and we were shy or hesitant; but she was very encouraging. She made us feel that it is ok and we can open up. She gave me the courage to speak openly about my feelings. She appreciated us all to be more involved and this made us open and be more communicative to others. (ID12)

### Participation in group sessions

A group-based intervention was appreciated and supported by nearly all the participants. They felt that the group allowed them to share information, and understand and explore solutions to their problems from each other’s perspectives. The mothers felt that the group was the only platform where they had an opportunity to express their emotions and feelings in a safe environment and this is something they are often not able to do with their own families.

… the best thing was I did not know anyone;. Sometimes you don’t want to discuss your personal matters with people you know. (ID13)

However, some participants felt reluctant to disclose personal issues in group settings due to fear of breach of confidentiality. It was suggested by some of the participants to incorporate some individual sessions in between group sessions.

In individual sessions, one can talk about problems which can’t be discussed in a group. And for groups we can share and learn from each other. (ID12)

Open discussions were nonetheless much favoured within the group as these offered flexibility along with giving the opportunity to practise assertiveness skills they had acquired during the sessions. Some participants preferred storytelling activities, which allowed them to talk about issues and explore solutions indirectly, without attracting attention.

I think open discussion was good and felt discussions helped in overcoming my anxieties and shyness. To me, these open discussions gave me the confidence to interact and overcome my depression. (ID8)

### Feeling confident and empowered

All the sessions were found useful by the participants as they reported overall improved wellbeing. They felt better able to deal with their day-to-day tasks. They also reported enhanced self-esteem, being more proactive, and being able to manage stress more effectively. They also spoke about embracing and adopting a positive view of life.

I am more relaxed and confident. Earlier, I could not speak and even go out of my house; now I go out with my friends and feel that I can learn new things as well. (ID6)

Learning positive thinking strategies and coping skills, along with group participation and a good relationship with the facilitator were reported as the most important factors for facilitating change.

Um…it’s the classes and the therapist. My life segments are still there but I have changed my thought process. I have a coping strategy for my problems. (ID10)

Most of the women reported being able to manage their everyday life more easily than before. The overall change was noted when dealing with children, family members, and oneself. Coping skills incorporated techniques for stress and time management as well as relaxation techniques.

Yes, it has. I am managing my family with less stress; I have realized and have gained the knowledge of overcoming my tension when I am dealing with my children and husband. I tell them I can do only so much at a time and they shouldn’t be expecting a lot from me. (ID2)

### Suggestions for improvement

A majority of the participants highlighted the need for such interventions to be ongoing as according to them psychosocial problems are deep-rooted and require much longer interventions.

Well, because it takes time to recover and then I might encounter some more issues in my life for which I might need guidance and assistance. You know, we are alone here; there is no one with whom to share and discuss our issues. (ID6)

Some participants suggested follow-up or ‘top-up’ sessions to motivate them to continue using the strategies learnt in the sessions and also as an opportunity to discuss additional issues and concerns with the facilitator.

Well, I thought the sessions went by too quickly and 12 weeks were not enough. I would have liked more sessions later because if something was missed or someone did not pick up something, at least they could have gone back and asked. (ID14)

One of the participants indicated health professionals’ lack of awareness of issues specific to ethnic minority communities and suggested that professionals such as GPs and nurse practitioners should be given culture-specific training.

Yes, what happened was, when I went to see my nurse um…they did not give me any direction where to go. I think GPs should be more involved, but these sessions were really good. (ID13)

## Discussion

The findings of our study highlight the experiences and acceptability of a culturally-adapted intervention (PHP) by British South Asian women. The main reasons for participants to engage and participate in the PHP were to gain support and to improve their self esteem and well-being. These findings are in line with the Villegas & McKay [30] study where low self-esteem has been reported as a risk factor for developing postnatal depression. The perception of the group as a way of getting social support by most of the participants is consistent with the findings from a US-based study reporting women who continued to be depressed two years after giving birth as more likely to lack social support [31].

The participants in our study reported feeling positive after the PHP intervention, although some faced barriers from the family. A key factor in retaining women in the PHP intervention was engaging with their family members, which facilitated their attendance at the sessions. A study with Pakistani women with postnatal depression living in Pakistan [32] reported that the intervention, while focusing on the mother and the infant, should also involve other members of the family for the mother to receive continued support so that she is able to engage with the intervention. An earlier study with British Pakistani women [10] reported that a major hindrance to social group participation was resistance from family members, particularly husbands.

A factor that helped with participation was the availability of free childcare to the participants at the intervention venue. In addition, some of the participants had older children; therefore, intervention sessions took place during term times. The Reay et al. [33] pilot study of group interpersonal psychotherapy for postnatal depression and research in the US [34] consider these to be key factors in improving engagement, particularly with hard-to-reach communities. Addressing transport arrangements was also reported to be an important factor in this study. The findings are also supported by Crockett et al.’s [35] study where lack of transport was found to be a barrier and women reported facing obstacles in getting transportation to the sessions. Chaudhry et al. [11] and later Gater et al. [17] also reported that provision of transport was an absolute necessity to ensure attendance to the intervention sessions by participants.

Furthermore, other difficulties raised by some participants were the inability to read and understand handouts written in English and the carrying out of between-session work due to personal and domestic commitments. There were, therefore, suggestions to add material in Urdu along with English, and some participants reported that completing the between-session work at home was not always a practical option for them due to time constraints. This needs to be considered in future research.

All the participants experienced an overall positive change in their attitudes, behaviours, and confidence. This positive change was attributed to a combination of facilitator input and coping strategies acquired over the 12 weekly sessions. The role of the facilitator was appreciated highly and the participants found the facilitator to be culturally aware and appropriately trained. The facilitator was open and communicative and this enhanced the overall process and led to increased positivity in the participants. Rahman [32] states that an understanding of the sociocultural context is essential for culturally-adapted interventions. In Rahman’s [32] study the community health workers were from the same community as the women, and understood the sociocultural context of the women’s problems which was found to improve engagement.

Because the PHP intervention was group-based, all the participants found factors such as sharing information, relating problems with each other, and gaining from the experiences of others helpful, and these contributed to their overall positive feelings. This is similar to the findings of Chaudhry et al. [14] and Gater et al. [10]. Some of the participants highlighted the need for some individual sessions along with the group sessions. They felt the need to talk about private matters to the facilitator on a one-to-one basis.

The impact of the PHP intervention was reflected in an overall positive change in participants’ dealing with family members and children. Particularly participants reported feeling calmer and much more relaxed when dealing with their children.

### Strengths and limitations of the study

A major strength of this study is the recruitment of participants from a hard-to-reach British South Asian community—Pakistani, Bangladeshi, and Indian women. An additional strength is the ability to engage depressed women during the postnatal period for up to 6 months, for follow-up assessments, and for interviews. Another strength of the study is the use of bilingual researchers which allowed participants to complete the interviews in their preferred language. This is one of very few studies looking at the overall process of development and implementation of culturally-specific psychological interventions for British South Asian women.

This study took place in the North West region of England; therefore, these results may not be generalizable to other regions in the UK. Most of the participants were of first-generation South Asian origin living in England. These participants were generally isolated and faced additional social pressures. Another limitation of this study is the lack of availability of information from mothers who dropped out and did not attend any of the sessions. This information would have contributed to a deeper understanding of the acceptability of PHP intervention. Further limitation of this study is the conservative definition of completers which was 4 or more sessions out of 12 sessions. This is a conservative value because the mean number of sessions was 6. Another limitation of the study is the lack of inquiry into the impact of intimate partner or other forms of family violence on postnatal mental health problems among the participants. The intervention appears not to have focused on empowerment or other rights-based strategies. Since some of the quotes by the participants suggest coercion and control, further studies are required to gain a clearer understanding of these challenges.

## Conclusions

The results suggest that this culturally-adapted psychological intervention is acceptable to British South Asian women. Furthermore, it appears from this qualitative study that culturally-sensitive psychological interventions can lead to better health outcomes and higher overall satisfaction levels. Interventions targeting postnatal depression in South Asian women should pay particular attention to ways of improving social support, independent coping strategies, involvement of the culturally-sensitive facilitator, language aspects, childcare and transport support, and group discussion techniques. Future research should consider including individual sessions in between group sessions to further improve engagement and perhaps health and social outcomes as well.
